# DNA Methylation-Mediated Downregulation of *DEFB1* in Prostate Cancer Cells

**DOI:** 10.1371/journal.pone.0166664

**Published:** 2016-11-11

**Authors:** Jaehyouk Lee, Jun Hyun Han, Ara Jang, Jin Wook Kim, Soon Auck Hong, Soon Chul Myung

**Affiliations:** 1 Department of Urology, Chung-Ang University College of Medicine, Seoul 06974, Republic of Korea; 2 Advanced Urogenital Diseases Research Center, Chung-Ang University College of Medicine, Seoul 06974, Republic of Korea; 3 Bio-Integration Research Center for Nutra-Pharmaceutical Epigenetics, Chung-Ang University, Seoul 06974, Republic of Korea; 4 Department of Urology, Hallym University Dongtan Sacred Heart Hospital, Hwaseong-si 18450, Republic of Korea; 5 Department of Pathology, Soonchunhyang University College of Medicine, Cheonan 31151, Republic of Korea; University of Navarra, SPAIN

## Abstract

Epigenetic aberrations play crucial roles in prostate cancer (PCa) development and progression. The *DEFB1* gene, which encodes human ß-defensin-1 (HBD-1), contributes to innate immune responses and functions as a potential tumor suppressor in urological cancers. We investigated whether differential DNA methylation at the low CpG-content promoter (LCP) of *DEFB1* was associated with transcriptional regulation of *DEFB1* in PCa cells. To identify distinct CpG loci within the *DEFB1* LCP related to the epigenetic regulation of *DEFB1*, we performed an *in vitro* methylated reporter assay followed by bisulfite sequencing of the *DEFB1* promoter fragment. The methylation status of two adjacent CpG loci in the *DEFB1* LCP was found to be important for *DEFB1* expression in PCa cells. Paired epithelial specimens of PCa patients (*n* = 60), which were distinguished as non-tumor and tumor tissues by microdissection, were analyzed by bisulfite pyrosequencing of site-specific CpG dinucleotide units in the *DEFB1* LCP. CpG methylation frequencies in the *DEFB1* LCP were significantly higher in malignant tissues than in adjacent benign tissues across almost all PCa patients. These results suggested that methylation status of each CpG site in the *DEFB1* promoter could mediate downregulation of *DEFB1* in PCa cells.

## Introduction

Prostate cancer (PCa) represents one of the most common non-cutaneous malignant neoplasms in men worldwide [1,2]. In a recent study, it was proposed that PCa can serve as a model for “epigenetic catastrophe” in which epigenetic changes occurring during the earliest stages of tumor initiation are maintained throughout its progression [3]. DNA methylation has been shown to play a critical role in regulating the expression of key genes involved in prostate morphogenesis [4]. Alterations in DNA methylation occur with aging, which is a major risk factor for PCa [5], and age-induced aberration in the DNA methylome has been implicated in prostate carcinogenesis [6,7]. DNA hypermethylation is the most extensively characterized epigenetic alteration occurring in patients with PCa [2]. Tumor suppressor genes silenced by promoter hypermethylation in PCa are involved in important cellular processes, including cell cycle control, apoptosis, DNA damage repair and hormone response [8]. DNA methylation-based biomarkers for PCa have been classified into early cancer detection, aggressiveness, and prognosis. Hypermethylation of a CpG island in the promoter of *GSTP1*, which encodes a detoxification enzyme, π-class glutathione S-transferase, is a molecular hallmark of PCa; it is present in ~70% of high-grade prostatic intraepithelial neoplasia (HG-PIN) lesions and in more than 90% of adenocarcinomas [3,9]. Further, methylation of *GSTP1* appears to perform better in combination with prostate-specific antigen (PSA) and other methylation biomarkers [7]. Remarkably, the *APC* gene was frequently found to be methylated in PCa [10], and methylation of the *RASSF1a* promoter was strongly correlated with an increased risk of PCa aggressiveness and tumor progression [11,12]. Notably, a combination of *GSTP1*, *RARß2*, *RASSF1a*, and *APC* has been the best at discriminating cancer from non-cancer specimens [13]. Multigene promoter methylation testing has been suggested to improve PCa sensitivity [14]. For example, the methylation status of *APC*, *GSTP1*, and *MDR1* could be used as a diagnostic and staging biomarker for PCa [15]. In addition, the prognostic potential of DNA methylation markers for PCa has been demonstrated in multiple studies [16]. Notably, the combined methylation profile of *CD44* and *PTGS2* was determined as a prognostic marker for biochemical recurrence following radical prostatectomy [17]. Furthermore, in the ‘post-PSA’ era, epigenetic field defects should be considered when analyzing heterogeneous and multifocal tissues of PCa.

Chronic infection or chronic inflammatory states may play a crucial role in the development of various human cancers [1,18]. Epidemiological and histopathological studies indicate that a history of prostatitis and sexually transmitted infections (STIs) increase the risk of PCa, suggesting that prostatic carcinogenesis may be triggered by inflammation [18,19]. Data from a recent study suggested that environmental factors, including infectious agents, can lead to the development of chronic inflammation and regenerative risk factor lesions such as proliferative inflammatory atrophy (PIA) in the prostate gland of humans [1]. PIA, which is considered a precursor of prostatic intraepithelial neoplasia (PIN) and PCa, often develops directly adjacent to these lesions [18]. It has also been postulated that PIA or proliferative atrophy is a manifestation of the “field effect” caused by environmental exposure [1].

Antimicrobial peptides (AMPs) play a key role in innate immune response against bacteria, viruses, fungi, and parasites [20,21]. Defensins are small, polycationic members of the AMP family, which includes α- and ß-defensins and cathelicidins. In the human genome, genes encoding various ß-defensins are located in a cluster on chromosome 8p23.1 [22]. Deletion of this 8p area is frequently observed in patients with PCa [23]. In addition to their direct antimicrobial clearance activities as part of the first-line defense mechanism, β-defensins function as both proinflammatory mediators and suppressors of immune responses [22]. The *DEFB1* gene, encoding human ß-defensin-1 (HBD-1), is unique because it is constitutively expressed in various epithelial tissues, including the skin and respiratory and urogenital tracts [24]. Cancer-specific downregulation of HBD-1 was observed in prostatic carcinomas [25]. Moreover, ectopic overexpression of *DEFB1* inhibits the proliferation of prostate, renal, and bladder cancer cells [23]. These findings suggested that *DEFB1* potentially functions as a tumor suppressor gene in urological cancers [23].

Here, we investigated whether the downregulation of *DEFB1* was associated with the methylation status of single CpG locus within the *DEFB1* LCP in PCa cells. To our knowledge, PCa-associated epigenetic abnormalities in genes lacking high CpG-content promoters (HCPs) have not been reported to date. The present study provided the evidence that the methylation of single CpG sites in the non-CpG island promoter was important for transcriptional regulation of *DEFB1* in PCa cells.

## Materials and Methods

### Ethics statement

This study was approved by the Institutional Review Board of the Chung-Ang University Hospital. All patients gave their written informed consent to participate in the study.

### Cell culture and reagents

The human prostate epithelial cell line HPEpiC was obtained from ScienCell Research Laboratories (Carlsbad, CA, USA), and the human PCa cell lines LNCaP, DU145, and PC-3 were purchased from the American Type Culture Collection (Manassas, VA, USA). HPEpiC cells were cultured in Prostate Epithelial Cell medium (ScienCell Research Laboratories), LNCaP cells were cultured in RPMI-1640 medium (WelGENE, Seoul, Korea), DU145 cells were cultured in Eagle’s minimum essential medium (WelGENE), and PC-3 cells were cultured in F-12K medium (WelGENE). Complete growth media for culturing PCa cell lines were supplemented with 10% fetal bovine serum (FBS), 100 IU/ml penicillin G, and 100 μg/ml streptomycin. All the cells were maintained at 37°C in a humidified incubator with an atmosphere of 5% CO_2_. PCa cell lines were treated with 2′-deoxy-5-azacytidine (DAC; Sigma-Aldrich, St. Louis, MO, USA) for 72 h.

### Tissue samples

PCa tissues isolated by radical prostatectomy (RP) were obtained from the Korea Prostate Bank (Seoul, Korea). For histopathological diagnoses of matched non-tumor and tumor tissues, 60 frozen tissue samples were sectioned and stained with hematoxylin and eosin (H&E). Malignant cells and adjacent benign cells were selectively procured from the H&E-stained slides by microdissection with a 30G1/2 hypodermic needle, as described previously [26]. DNA was extracted using a modified single-step DNA extraction method with proteinase K treatment [26]. Briefly, the procured cells in 30 μl of DNA extraction buffer [100 mM Tris-HCl (pH 8.0), 1% Tween 20, and 0.1 mg/ml proteinase K] were incubated at 52°C for 1−2 days. The mixture was boiled to inactivate proteinase K at 100°C for 10 min. The genomic DNA of microdissected samples was purified using the DNA Clean & Concentrator^™^-5 Kit (Zymo Research, Irvine, CA, USA) and was quantified using a spectrophotometer.

### Quantitative reverse transcription PCR (RT-PCR)

Total RNA was isolated from the cell lines using the RNeasy Mini Kit (Qiagen, Hilden, Germany), according to the manufacturer’s instructions. Next, cDNA was synthesized from 1 μg of total RNA using the ImProm-II^™^ Reverse Transcription System (Promega, Madison, WI, USA). The sequences of primer used for RT-PCR amplification of *DEFB1* cDNA are 5′-ATGAGAACTTCCTACCTTCTGCTGT-3′ (forward) and 5′-TGGTAAAGATCGGGTAGGCAGAA-3′ (reverse). The cycling conditions were as follows: initial denaturation at 94°C for 3 min; 33−35 cycles of denaturation at 94°C for 30 s, annealing at 65°C for 30 s, and extension at 72°C for 20 s; and a final extension at 72°C for 7 min. All amplifications were performed using AccuPower PCR PreMix (Bioneer, Daejeon, Korea). The human *ACTB* gene (encoding ß-actin) was amplified as an endogenous control.

Real-time cDNA synthesis was performed by conducting a two-step RT-PCR with 1 μg total RNA as the template, using the QuantiTect Reverse Transcription Kit (Qiagen). Next, 1 μl diluted cDNA was used as the template for PCR with the Rotor-Gene SYBR Green PCR Kit (Qiagen), according to the manufacturer’s instructions. Amplification and quantitative analysis were performed using the Rotor-Gene Q 5plex HRM system (Qiagen).

### Immunohistochemistry

Expression of HBD-1 in prostate cancer tissue samples was studied using the UltraVision LP Detection System (Lab Vision Corp., Fremont, CA, USA), as described previously[27]. Briefly, slides with prostate cancer tissue sections were dewaxed in xylene for more than 20 min and then sequentially rehydrated in 100%, 95%, 90%, and 80% ethanol solutions. After a 5-min rinse in water, the slides were pretreated with 0.01M sodium citrate buffer and autoclaved for 1 min at 121°C for antigen retrieval. After the slides were rinsed, endogenous peroxidase activity was blocked by treatment with 3% H_2_O_2_ for 30 min. A primary mouse polyclonal antibody against HBD-1 (1:50, Abcam, Cambridge, MA, USA) was applied to the sections on the slides, following which the sections were incubated for 2 h in a moist chamber at room temperature. Sections for negative control were incubated with the same reagents and underwent the same epitope retrieval protocol without the primary antibody (S1 Fig). After the slides were rinsed with PBS, they were incubated with secondary antibody for 10 min at room temperature and then rinsed in PBS. The slides were incubated with a horseradish peroxidase conjugated antibody for 10 min, rinsed in PBS, and incubated with diaminobenzidine for 10 min. After the slides were counterstained using Mayer’s hematoxylin, they were dehydrated and coverslipped.

### *In vitro* methylated reporter assay

A 790-bp fragment harboring 14 CpG sites located at the 5′-end region of *DEFB1*, a 5′ BamHI site, and a 3′ HindIII site was synthesized and cloned into the pCpGfree-basic-Lucia reporter plasmid (InvivoGen, San Diego, CA, USA) that codes for a secreted coelenterazine-utilizing variant of luciferase. Briefly, 5 μg of the Lucia reporter plasmid was methylated using 12 U of M.*SssI* CpG methyltransferase (New England BioLabs, Ipswich, MA, USA) *in vitro* at 37°C for 20 h. After purification with the DNA Clean & Concentrator^™^-5 Kit (Zymo Research) according to the manufacturer’s protocol, methylation of the plasmid was verified by bisulfite pyrosequencing of the selected CpG units in the *DEFB1* promoter (S2 Fig). Next, HEK293T cells were transiently transfected with unmethylated or methylated reporter plasmids by using the jetPEI reagent (Polyplus-transfection, New York, NY, USA). Luciferase activity of the Lucia reporter in the supernatant of transfected cells at 72 h after the transfection was measured using the QUANTI-Luc assay reagent (InvivoGen). In each experiment, the cells were cotransfected with pCMV-CLuc 2 Control Plasmid (New England BioLabs), which encoded a secreted variant of *Cypridina* luciferase, as a normalization control. Luminescence induced by *Cypridina* luciferase in the cell supernatant was determined using the BioLux *Cypridina* Luciferase Assay Kit (New England BioLabs). Reporter activity was normalized by calculating the ratio of Lucia to CLuc activities.

### DNA methylation analyses

For bisulfite genomic sequencing analysis, genomic DNA was extracted from the cell lines by using the QIAamp DNA Mini Kit (Qiagen). Bisulfite modification of the genomic DNA was performed using the EZ DNA Methylation-Lightning^™^ Kit (Zymo Research), according to the manufacturer’s instructions. The bisulfite-converted genomic DNA was amplified using primer sets specific to the proximal promoter region of *DEFB1* (nucleotides -624 to -120 bp upstream of the transcription start site (TSS), which was denoted as +1) containing 6 CpG units. The sequences of the PCR primers used were as follows: forward (5′-TTGGTAGGGTTGAAGTGGGAG-3′) and reverse (5′- TAAAACCCTAATACCAACTCCTC-3′). Cycling conditions were as follows: initial denaturation at 94°C for 10 min; 45−50 cycles of denaturation at 94°C for 30 s, annealing at 52°C for 30 s, and extension at 72°C for 30 s; and a final extension at 72°C for 10 min. PCR products were purified and subcloned into the pGEM-T Easy Vector (Promega) for subsequent sequencing reactions. The nucleotide sequences of 20−25 independent clones were analyzed.

Further, the bisulfite-modified genomic DNA obtained from patients with PCa was amplified using primer sets specific to the *DEFB1* LCP. The sequences of the PCR primers used were as follows: forward (5′-TTTTTGTAAGGGAAGAGGGTGAAG-3′), 5′-biotinylated reverse (5′-TCACACTAAAATCCCTCCTTCTAAATCAC-3′), and sequencing (5′-AATTAAAGAGGTTAATATTAGTT-3′). Pyrosequencing reactions were performed using the PyroMark Gold Q96 Reagents (Qiagen) and quantitative analysis was performed using the PyroMark Q96 ID platform (Qiagen), according to the supplier’s instructions.

### Statistical analysis

Data from the quantitative RT-PCR and luciferase assay were analyzed using the Student’s *t* test. Significant differences with respect to the quantitative methylation data obtained from the pyrosequencing measurements between non-tumor and tumor tissue samples were determined using the Wilcoxon signed-rank test. A *P*-value of < 0.05 was considered statistically significant. All statistical analyses were conducted using IBM SPSS Statistics 20 (IBM Corp., Armonk, NY, USA).

## Results

### Specific single CpG dinucleotide sites are important for DNA methylation-mediated downregulation of *DEFB1* in PCa cells

**Fig 1:**
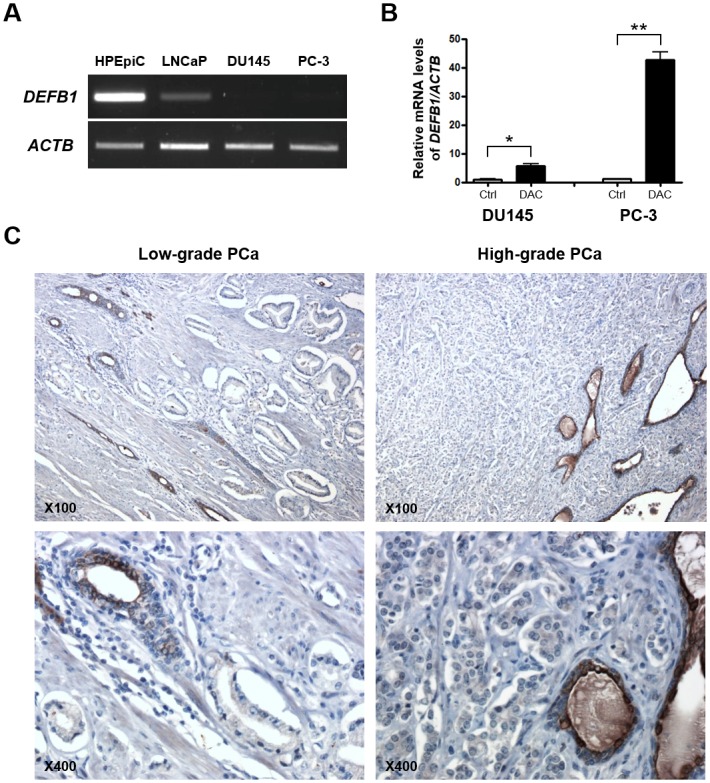
Downregulation of the *DEFB1* gene in prostate cancer (PCa) cell lines and tissues. (A) *DEFB1* mRNA expression in prostate epithelial cell lines was assessed by performing RT-PCR. The human *ACTB* gene (encoding β-actin) was amplified as an endogenous control. (B) Quantitative PCR was performed to evaluate the restoration of *DEFB1* mRNA expression in DU145 and PC-3 cells after treatment with DNA demethylating agent 2′-deoxy-5-azacytidine (DAC). *P < 0.05 and **P < 0.01. (C) Immunohistochemical staining of DEFB1 in prostate cancer tissue samples. PCa, prostate cancer; NT, non-tumor; T, tumor.

To investigate whether epigenetic silencing of *DEFB1* occurred in PCa, we first examined the mRNA levels of *DEFB1* in the human prostate epithelial cell line HPEpiC and the three PCa cell lines (Fig 1A). The RT-PCR results showed that *DEFB1* transcription was prominent in HPEpiC cells but was slightly low in LNCaP cells. Because *DEFB1* transcription seemed to be silenced in the PCa cell lines DU145 and PC-3, we determined whether the loss of *DEFB1* expression in these cells was associated with DNA methylation. Quantitative PCR was performed to examine the transcriptional restoration of *DEFB1* in DU145 and PC-3 cells after treatment with the DNA methyltransferase inhibitor 2′-deoxy-5-azacytidine (DAC) (Fig 1B). DAC-induced DNA demethylation significantly increased *DEFB1* mRNA expression in both DU145 and PC-3 cells (by 5.5- and 39.3-fold, respectively), implying that the loss of *DEFB1* expression in the PCa cell lines correlated with epigenetic inactivation involving DNA methylation. HBD-1 expression is universally detected in normal prostate tissues but is frequently lost in prostatic intraepithelial neoplasm (PIN), while 82% of PCa specimens exhibited either complete loss or only minimal expression of HBD-1 [25]. Immunohistochemical analysis of the HBD-1 protein in PCa sections in the present study confirmed the tumor-specific loss of HBD-1 expression (Fig 1C).

**Fig 2:**
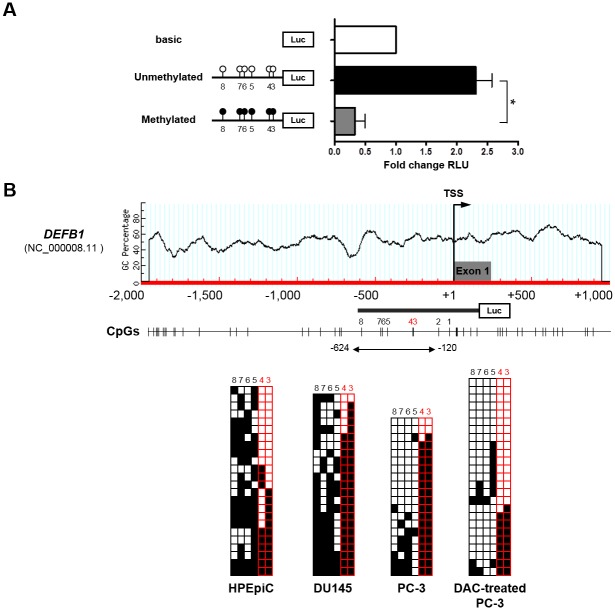
DNA methylation analyses of the low CpG-content promoter (LCP) of *DEFB1* in prostate cancer cell lines. (A) Promoter reporter assay of the 5′-end region of *DEFB1* in HEK293T cells using the pCpGfree-basicLucia reporter plasmid. The synthesized fragment contained 14 CpG dinucleotide sites (represented by lollipops). Of these, 8 CpGs were previously studied and are not shown here. (B) Bisulfite sequencing analysis of the *DEFB1* promoter harboring CpG 3−CpG 8 sites in prostate epithelial cell lines. Columns represent single CpG sites, and each row represents the DNA sequence of an individual clone. Unmethylated and methylated CpG units are depicted as white and black boxes, respectively.

To determine whether DNA methylation in the 5′-end region of *DEFB1* was important for modulating the transcriptional activity of the core promoter, we conducted an *in vitro* methylated reporter assay (Fig 2A). Because a previous study demonstrated a region critical for transcriptional activity of the *DEFB1* promoter [23], we synthesized a *DEFB1* fragment (NC_000008.11; −612 to +165 bp, relative to the TSS) containing the core promoter region, exon 1, and 14 CpG sites (Fig 2B). *In vitro* methylation was performed after cloning this fragment into a CpG-free luciferase plasmid to assess the role of CpG methylation in *DEFB1* transcription. HEK293T cells transfected with the methylated *HBD-1* reporter plasmid showed markedly decreased luciferase activity compared with cells transfected with unmethylated *DEFB1* plasmid. Moreover, methylation of the 14 CpG dinucleotide units in the 5′-end region of *DEFB1* decreased its promoter activity by approximately 86%, suggesting that DNA methylation directly affected its transcriptional regulation.

To identify site-specific CpG dinucleotide units critical for *DEFB1* expression in PCa cell lines, we performed bisulfite genomic sequencing of the proximal region upstream of the *DEFB1* LCP. Notably, a CpG island plot showed that the 5′-end region proximal to the *HBD-1* TSS had low GC content and was depleted at CpG loci (Fig 2B). A previous study provided evidence that cancer-specific methylation was not observed in both the proximal core promoter (i.e., CpG 1 and CpG 2 units; the first CpG dinucleotide located 31-bp upstream of the *DEFB1* TSS being assigned as CpG 1) and exon 1 (containing 6 CpG sites) in the 5′-end region of *DEFB1* in PCa cell lines and in normal and malignant prostate tissues [23]. Therefore, we determined the methylation profiles of the remaining 6 CpG dinucleotide units (i.e., CpG 3−CpG 8) that were initially included in the *in vitro* methylated reporter assay (Fig 2B). The methylation profile of the *DEFB1* LCP showed a significant difference in the methylation frequency of both the CpG 3 and CpG 4 units in HPEpiC cells compared with that in DU145 and PC-3 cells. While the region harboring the CpG 3 and CpG 4 dinucleotide units in HPEpiC cells, which showed active *DEFB1* transcription, exhibited relatively low DNA methylation levels, the same region in DU145 and PC-3 cells, which lacked *DEFB1* transcription, showed uniformly high DNA methylation levels. Further, bisulfite sequencing of the *DEFB1* LCP in the DAC-treated PC-3 cells indicated that these single CpG units were preferentially affected by the DNA demethylating agent DAC (Fig 2B), which was consistent with the above findings. Thus, our results suggested that the differentially methylated CpG 3 and CpG 4 units in the *DEFB1* LCP were particularly important for regulating *DEFB1* transcription in PCa cells.

### Methylation frequencies of two single CpG sites in the *DEFB1* LCP were higher in tumor tissues than in non-tumor tissues of PCa patients

To determine the distinct epigenotype of *DEFB1* in a series of matched PCa specimens, we focused on the epialleles of the *DEFB1* LCP containing the CpG 3 and CpG 4 units (Fig 2B) and compared the methylation frequencies of these two adjacent CpG sites in the microdissected samples. Table 1 presents the 60 matched tissue samples obtained from patients with PCa. The samples were classified primarily into the following four groups on the basis of their Gleason scores: GS ≤ 6; GS = 7 (3 + 4); GS = 7 (4 + 3); and GS ≥ 8.

**Table 1:** Clinical Characteristics of Patients with PCa.

Characteristics	*n*
**Age**, yrs	
Median (range)	66.5 (54–76)
**PSA**, ng/ml	
Median (range)	7.79 (1.55–90.3)
**Gleason score**	
≤ 6	15 (25%)
7 (3 + 4)	15 (25%)
7 (4 + 3)	15 (25%)
≥ 8	15 (25%)
**T stage**	
T2	25 (42%)
T3a	21 (35%)
T3b	14 (23%)

**Fig 3:**
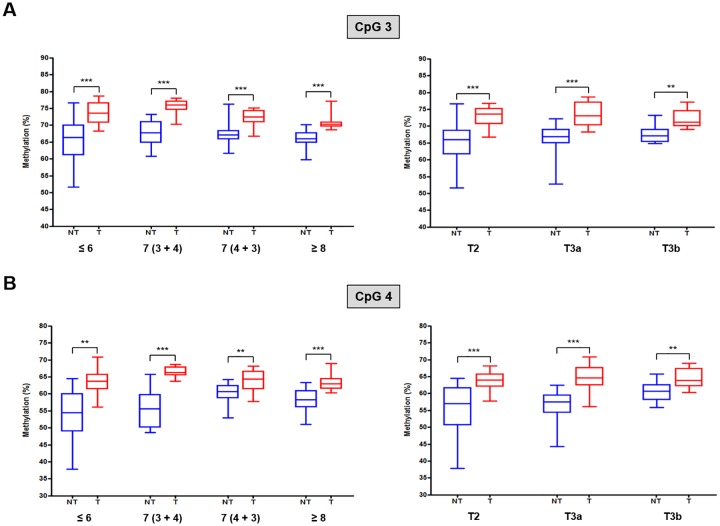
Association of the methylation frequencies of the (A) CpG 3 and (B) CpG 4 sites in the *DEFB1* LCP with the corresponding Gleason scores and T stages of PCa tissues. Data shown in the blue box plots represent the methylation levels in non-tumor tissue samples, and data shown in the red box plots represent the methylation levels in tumor tissue samples. NT, non-tumor; T, tumor. **P < 0.01 and ***P < 0.001.

Each group included 15 pairs of matched samples, and each bisulfite pyrosequencing experiment was performed in triplicate. Almost all the methylation frequencies of the two CpG sites in the *DEFB1* LCP were significantly higher in malignant tissues (i.e., T; tumor) than in the adjacent benign tissues (i.e., NT; non-tumor) examined, which was indicative of the tumor suppressor gene (Fig 3).

The highest significant difference was observed in the methylation frequency of the CpG 3 unit in the *DEFB1* LCP. Similar results were obtained for both the CpG units in the *DEFB1* LCP in malignant and adjacent benign tissues in patients with PCa classified according to the T stage. Thus, our results suggested that alterations in the DNA methylation status of these two differentially methylated CpG units in the *DEFB1* LCP might be associated with malignant prostatic epithelium.

## Discussion

Previous studies have been conducted in attempt to elucidate possible mechanisms underlying DNA methylation-dependent regulation of *DEFB1* in patients with PCa [23] and chronic obstructive pulmonary disease (COPD) [28]. However, no significant differences were observed in the methylation statuses of CpG sites in the *DEFB1* LCP among different PCa cell lines, between normal and malignant prostate tissues, and between patients with COPD and controls. Because the 5′-end region of *DEFB1* is devoid of any defined CpG island (length, ≥ 200 bp; GC content, ≥ 50%; and Obs_CpG_/Exp_CpG_, ≥ 0.6) [29], previous studies might not have analyzed subtle differences in the methylation patterns of the limited number of CpG loci that are important for *DEFB1* transcription. Here, our findings suggested that differentially methylated single CpG sites within the non-CpG island promoter of *DEFB1* might be correlated with transcriptional regulation of *DEFB1* in PCa cells. In addition, relatively small differences were observed in the methylation levels of each CpG unit in the *DEFB1* LCP between malignant lesions and the neighboring benign tissues. This result might be explained in part by the finding that HCPs linked to CpG islands are predominantly hypomethylated, whereas LCPs, which do not contain CpG islands, are primarily methylated [30]. Moreover, each individual CpG locus of *DEFB1* may be assigned to methylation variable position (MVP) candidates, which can be considered as the epigenetic equivalent of single nucleotide polymorphisms [31].

Promoter methylation patterns in specific genes have emerged as important biomarkers for prostate carcinogenesis [32,33]. DNA methylation-based PCa biomarkers could be chosen for cancer detection in non-invasive screening using biofluids (e.g., urine, ejaculate, and expressed prostate secretion), ancillary tools for histopathological observation for diagnosis following short term re-biopsy, pre-therapeutic prediction of recurrence and progression, and assessment of prognosis [2,7]. Intriguingly, multiple biomarkers support the presence of the “field effect,” which can be observed during PCa progression, when the cancer develops in multifocal areas and when neoplastic or pre-neoplastic cells are present in histologically normal fields proximal to cancer tissues [34,35]. For instance, *GSTP1* hypermethylation was more confined to PCa cells, whereas *APC* and *RARß2* were frequently methylated in adjacent normal tissues as well as in HG-PIN lesions [36]. Because DNA methylation markers are being commercially utilized in PCa management, additional studies on epigenetic alterations of *DEFB1* in PCa development and progression will provide promising potential for *DEFB1* as a new PCa-associated biomarker candidate.

## Conclusion

We identified specific epialleles of *DEFB1* in PCa cells that represented hypomethylated single CpG sites in *DEFB1*-expressing cells and hypermethylated single CpG sites in cells lacking *DEFB1* expression. Our data suggested that DNA methylation pattern of the non-CpG island promoter of *DEFB1* might affect epigenetic silencing of *DEFB1* in PCa cells. Consistent with the possible mechanism underlying the “field effect,” which suggests the progressive accumulation of methylated copies of genes such as *APC* and *RARß2*, we hypothesized that alteration of the DNA methylation status in the *DEFB1* LCP could help in diagnosing PCa.

## Supporting Information

S1 Fig Negative control of IHC experiment in Fig 1C. (TIF)

S2 Fig Quantitative methylation analysis of the unmethylated and *in vitro* methylated luciferase reporter plasmids. Four CpG dinucleotide sites located in the *DEFB1* promoter were assessed by bisulfite pyrosequencing: white and black boxes; unmethylated and methylated CpG sites, respectively.
(TIF)

## Abbreviations

AMP: antimicrobial peptides
APC: adenomatous polyposis coli
DAC: 2′-deoxy-5-azacytidine
GSTP1: glutathione S-transferase pi 1
HBD-1: human ß-defensin-1
LCP: low CpG-content promoter
PCa: prostate cancer
PSA: prostate-specific antigen
RARß2: retinoic acid receptor ß2
RASSF1a: ras association domain family protein 1 isoform A
TSS: transcription start site
