# Supplementary figures and images for: DNA Methylation-Mediated Downregulation of DEFB1 in Prostate Cancer Cells
Source: PLoS One. 2016 Nov 11;11(11):e0166664. doi: 10.1371/journal.pone.0166664 (PMC5105953; doi:10.1371/journal.pone.0166664)

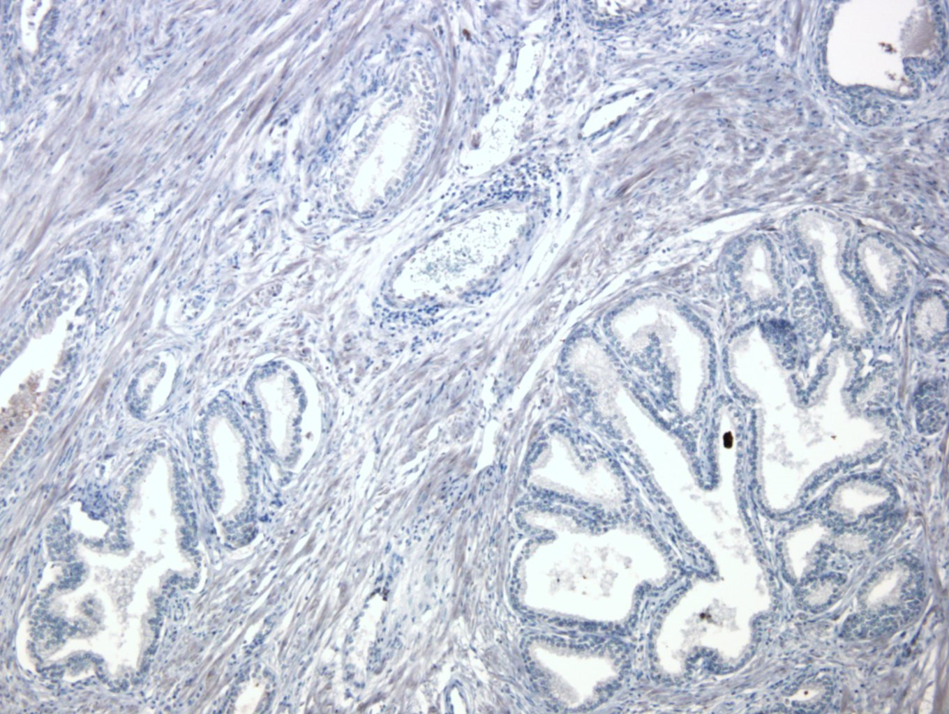

Supplement: S1 Fig — (TIF) [file pone.0166664.s001.tif]

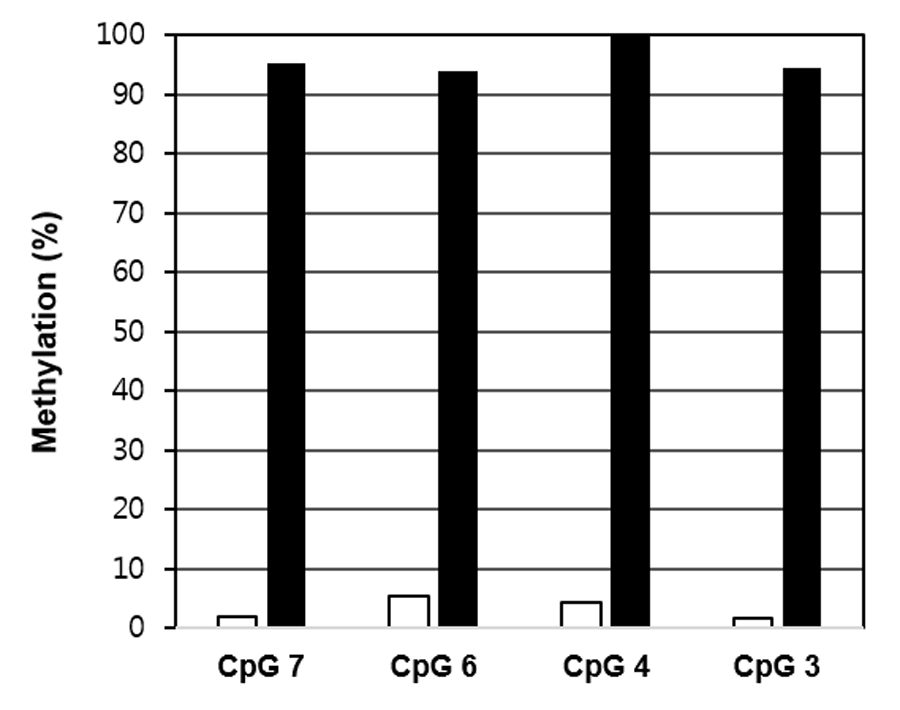

Supplement: S2 Fig — Four CpG dinucleotide sites located in the DEFB1 promoter were assessed by bisulfite pyrosequencing: white and black boxes; unmethylated and methylated CpG sites, respectively. (TIF) [file pone.0166664.s002.tif]
